# Labeling of Multiple HIV-1 Proteins with the Biarsenical-Tetracysteine System

**DOI:** 10.1371/journal.pone.0017016

**Published:** 2011-02-11

**Authors:** Cândida F. Pereira, Paula C. Ellenberg, Kate L. Jones, Tara L. Fernandez, Redmond P. Smyth, David J. Hawkes, Marcel Hijnen, Valérie Vivet-Boudou, Roland Marquet, Iain Johnson, Johnson Mak

**Affiliations:** 1 Centre for Virology, Burnet Institute, Melbourne, Victoria, Australia; 2 Monash Micro Imaging, Monash University, Clayton, Victoria, Australia; 3 Department of Medicine, Monash University, Clayton, Victoria, Australia; 4 Department of Biochemistry and Molecular Biology, Monash University, Clayton, Victoria, Australia; 5 Department of Microbiology, Monash University, Clayton, Victoria, Australia; 6 Architecture et Réactivité de l'ARN, Université de Strasbourg, CNRS, IBMC, Strasbourg, France; 7 Life Technologies Corporation, Eugene, Oregon, United States of America; Louisiana State University Health Sciences Center, United States of America

## Abstract

Due to its small size and versatility, the biarsenical-tetracysteine system is an attractive way to label viral proteins for live cell imaging. This study describes the genetic labeling of the human immunodeficiency virus type 1 (HIV-1) structural proteins (matrix, capsid and nucleocapsid), enzymes (protease, reverse transcriptase, RNAse H and integrase) and envelope glycoprotein 120 with a tetracysteine tag in the context of a full-length virus. We measure the impact of these modifications on the natural virus infection and, most importantly, present the first infectious HIV-1 construct containing a fluorescently-labeled nucleocapsid protein. Furthermore, due to the high background levels normally associated with the labeling of tetracysteine-tagged proteins we have also optimized a metabolic labeling system that produces infectious virus containing the natural envelope glycoproteins and specifically labeled tetracysteine-tagged proteins that can easily be detected after virus infection of T-lymphocytes. This approach can be adapted to other viral systems for the visualization of the interplay between virus and host cell during infection.

## Introduction

Fluorescent viral fusion proteins have been instrumental in the visualization of the intracellular behavior of viruses during infection [1]–[3]. However, the incorporation of these 27 kDa fluorescent proteins (FPs) into most viral proteins has limited application due to its potential impact on viral protein function and infectivity [4]–[7]. This problem has been partially overcome with the development of the biarsenical-tetracysteine labeling system [8], in which the protein of interest is fused to a six to twelve amino acids tetracysteine motif (TC tag), which is only 0.5–1 kDa and therefore less likely to interfere with the structure or biological activity of the protein. The TC tag can be labeled with membrane-permeable biarsenical dyes such as FlAsH and ReAsH [9], [10].

The TC tag has been inserted at the carboxy-terminus of the human immunodeficiency virus type 1 (HIV-1) matrix (MA) [11], [12], HIV-1 integrase (IN) [13] and vesicular stomatitis virus (VSV) MA protein [5], [14] without compromising virus infectivity. However, insertion of the TC tag into the cyclophilin-binding loop of HIV-1 capsid (CA) severely impaired infectivity to the extent that virus infectivity needed to be rescued by co-expression of TC tagged and wild-type (wt) proteins [15]. Another recent study has elegantly shown that the loop/linker regions, but not the α-helix regions of influenza A virus non-structural protein 1, can accommodate TC tags without affecting virus infectivity [16].

Two major limitations of the biarsenical-tetracysteine system are the high degree of background fluorescence [17], [18], and the fact that the target cysteines in the TC tag must be in their reduced form prior to binding to the biarsenical dyes [12], [19]. Therefore, previous studies of TC tagged viruses have resorted to: (a) the use of a higher contrast twelve amino acids TC motif [12], (b) extracellular labeling of TC tagged proteins under acutely reducing conditions [12], [13] and/or (c) increasing the number of available TC tagged proteins inside the target cell via pseudotyped viruses to improve the signal to noise ratio [11]–[13], [15]. In the case of HIV-1, HIV-1 pseudotyped with VSV glycoprotein (VSV-G) instead of its natural envelope (Env) glycoproteins is commonly used to increase the number of HIV-1 proteins inside target cells. However, VSV-G pseudotyping does not represent the natural viral entry process for HIV-1. Furthermore, the extracellular labeling of viruses with biarsenical dyes under highly reducing conditions is not suitable for all virus systems [14].

The aim of the present study was to specifically label all of the structural proteins and enzymes of HIV-1 with a TC tag in the context of a full-length virus without affecting virus infectivity. We employed three different strategies in this study. Firstly, we inserted a six amino acids TC tag upstream and/or downstream of each of the protease cleavage sites in the Gag or Gag-Pol precursor protein. This resulted in specific labeling of MA, CA, nucleocapsid (NC), protease (PR), reverse transcriptase (RT), RNase-H (RN) and IN. Secondly, we inserted the TC tag into two highly flexible regions of the mature RT enzyme and the Env glycoprotein 120 (gp120). Thirdly, using a conditional complementation rescue approach, we generated an HIV-1 construct containing 80% TC-labeled NC proteins. We also show that virus production in the presence of biarsenical dyes followed by the removal of unbound dye is a highly specific method for the labeling of viral proteins containing a TC tag.

## Results

### Generation of mutant viruses containing tetracysteine-tagged proteins

The aim of this study was to specifically label all of the structural proteins and enzymes of HIV-1 with a TC tag in the context of a full-length virus without affecting virus infectivity. To this end, we created several full-length HIV-1 constructs containing TC tagged proteins (HIV^TC^). To minimize the potential impediment on virus infectivity, we used the six amino acids TC motif instead of the higher contrast twelve amino acids TC motif [10].

**Figure 1:**
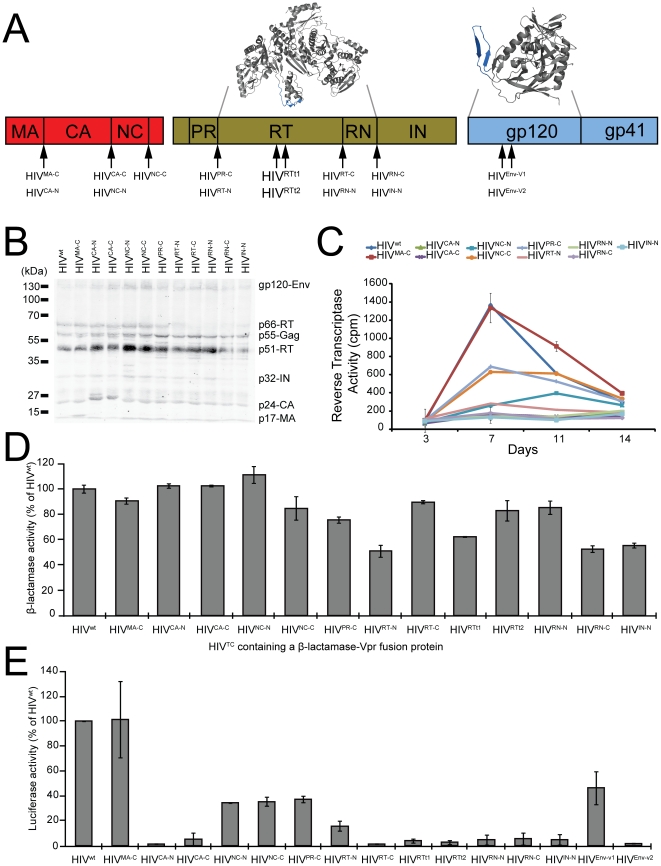
Genetic labeling of HIV-1 proteins and its impact on virus infectivity. (A) Diagram of the HIV-1 *gag* (red), *pol* (brown) and *env* (blue) open reading frames and the positions where the tetracysteine (TC) tag motif was inserted. The majority of the TC motifs were inserted five amino acids downstream (-N tags) or upstream (-C tags) of the protease cleavage site and the first or last five amino acids of the viral protein coding sequence were repeated to prevent alterations in the minimal recognition site for the viral protease. The left insert shows the crystal structure of the RT enzyme and the positions in the “thumb” region where the TC tag was inserted are shown in blue. The right insert shows the crystal structure of the Env gp120 and the positions in the V1/V2 region where the TC tag was inserted are shown in blue. (B) Virion protein processing profiles of 293T cell-derived HIV^MA-C^, HIV^CA-N^, HIV^CA-C^, HIV^NC-N^, HIV^NC-C^, HIV^PR-C^, HIV^RT-N^, HIV^RT-C^, HIV^RN-N^, HIV^RN-C^ and HIV^IN-N^ (lanes 1-12, respectively). HIV-1 proteins were detected by Western Blot using pooled HIV-positive patient sera. Data are representative of 2 independent experiments. (C) The infectivity of HIV^MA-C^, HIV^CA-N^, HIV^CA-C^, HIV^NC-N^, HIV^NC-C^, HIV^PR-C^, HIV^RT-N^, HIV^RN-N^, HIV^RN-C^ and HIV^IN-N^ was assessed by comparing their replication kinetics in peripheral blood mononuclear cells with HIV^wt^. Samples were collected at 3, 7, 11 and 14 days post infection, and the levels of virus replication were monitored using an *in vitro* reverse transcriptase assay that is specific for HIV-1 enzymatic activity. Data are representative of 2-3 independent donors. Error bars represent technical replicates. (D) The capacity of several HIV^TC^ to enter target cells was assessed by measuring β-lactamase activity in MT-2 cells that have been infected with HIV^TC^ containing a β-lactamase-Vpr fusion protein. Error bars, s.d. are based on the averages of 2-3 independent experiments. (E) The capacity of HIV^TC^ to infect target cells was assessed by measuring luciferase activity in the indicator TZM-bl cells that have been infected with the indicated viruses. Error bars, s.d. are based on the averages of 2-3 independent experiments.

In the first labeling strategy we inserted the TC tag within the N-terminus or C-terminus of the major mature Gag and Pol proteins. The natural proteolytic cleavage sites were preserved by duplicating the first or last five amino acids of the viral protein coding sequences of the corresponding mature proteins (Figure 1A and Table 1). Using this strategy the following HIV^TC^ were generated: HIV^MA-C^, HIV^CA-N^, HIV^CA-C^, HIV^NC-N^, HIV^NC-C^, HIV^PR-C^, HIV^RT-N^, HIV^RT-C^, HIV^RN-N^, HIV^RN-C^ and HIV^IN-N^. The protein processing profiles of these HIV^TC^ were very similar from HIV^wt^ with a few exceptions. HIV^RT-N^, HIV^RT-C^, HIV^RN-N^, HIV^RN-C^ and HIV^IN-N^ have reduced levels or completely lack the p66-RT subunit (Figure 1B). The MA protein in HIV^MA-C^ showed a slower electrophoretic mobility, which is likely due to the additional six amino acids TC tag, as described previously [11], [12]. Similarly, the CA protein in HIV^CA-N^ and HIV^CA-C^ also showed a slower electrophoretic mobility and appeared as a double band in HIV^CA-N^. The appearance of the CA doublet may result from non-specific protease cleavage at the N-terminus of CA in HIV^CA-N^. To assess the effect of these modifications on the viral life-cycle, the replication kinetics of HIV^MA-C^, HIV^CA-N^, HIV^CA-C^, HIV^NC-N^, HIV^NC-C^, HIV^PR-C^, HIV^RT-N^, HIV^RN-N^, HIV^RN-C^ and HIV^IN-N^ were assessed in peripheral blood mononuclear cells (PBMCs), which are the natural target cells of HIV-1 (Figure 1C). HIV^MA-C^ replication levels and kinetics were very similar to HIV^wt^. HIV^NC-C^ and HIV^PR-C^ were 54% and 50% less infectious than HIV^wt^, respectively. The replication kinetics of HIV^NC-N^ was delayed (maximum RT activity at day 11). The remaining viruses were between 79-91% less infectious than HIV^wt^.

**Table 1:** Overview of the modifications introduced in the HIV-1 genome.

Construct	Amino acid sequence
	**Last 5 amino acids – AG linker (Nae I restriction site) – TC tag – Duplication of Last 5 amino acids**
HIV^MA-C^	VSQNYAGCCRECCVSQNY
HIV^CA-C^	KARVLAGCCRECCKARVL
HIV^NC-C^	ERQANAGCCRECCERQAN
HIV^PR-C^	CTLNFAGCCPGCCCTLNF
HIV^RT-C^	GAETFAGCCPGCCGAETF
HIV^RN-C^	IRKVLAGCCPGCCIRKVL
	**Duplication of First 5 amino acids –TC tag – AG linker (Nae I restriction site) – First 5 amino acids**
HIV^CA-N^	PIVQNCCRECCAGPIVQN
HIV^NC-N^	IQKGNCCRECCAGIQKGN
HIV^RT-N^	PISPICCPGCCAGPISPI
HIV^RN-N^	YVDGACCPGCCAGYVDGA
HIV^IN-N^	FLDGICCPGCCAGFLDGI
	**a.a. sequence – TC tag – a.a. sequence**
HIV^RTt1^	PIVLPEKCCPGCCDSWTV
HIV^RTt2^	KALTEVVCCPGCCPLTE
HIV^Env-V1^	CTDLKNDTNTCCPGCCNSSSGRMIMEKGEIKN
HIV^Env-V2^	SFNISTSIRDKVQKEYAFFYKLDIVPIDNCCPGCCTSYRLISC

In the second labeling strategy, we introduced the TC tag into flexible regions of the RT and Env in order to limit the structural and functional interference with the protein. These flexible regions were chosen based on the crystal structure and biochemical properties of the mature proteins (Figure 1A inserts). The TC tag was inserted into the RT “thumb” subdomain between β14 and αH or between αI and αJ to generate the HIV^RTt1^ and HIV^RTt2^, respectively. HIV^Env-V1^ and HIV^Env-V2^ contain a TC tag in the variable loops of the V1 or V2 region of gp120, respectively. The location of each TC tag is indicated in Table 1.

The capacity of HIV^TC^ to enter lymphoid cells was assessed by measuring the cytosolic activity of viral-core-associated β-lactamase [20]. All HIV^TC^ were able to enter lymphoid cells (Figure 1D), and all viruses showed similar entry efficiency as HIV^wt^, with the exception of HIV^RT-N^, HIV^Rt1^, HIV^RN-C^ and HIV^IN-N^, which were approximately 50% less efficient. The capacity of HIV^TC^ to infect the indicator cell line TZM-bl (Figure 1E) clearly showed that HIV^MA-C^ replicates as efficiently as HIV^wt^, as reported previously [11], [12]. The remaining viruses showed different degrees of infectivity. HIV^NC-N^, HIV^NC-C^, HIV^PR-C^ and HIV^Env-V1^ were 66%, 65%, 63% and 54% less infectious than HIV^wt^, respectively. HIV^RT-N^ was 84% less infectious than HIV^wt^ and the remaining viruses were approximately 95% less infectious than HIV^wt^.

### Infectivity rescue of mutant viruses containing tetracysteine-tagged nucleocapsid proteins

Since the structural proteins of HIV-1 are more permissive to the insertion of the TC tag we attempted to rescue the infectivity of the HIV^CA-N^, HIV^NC-N^ and HIV^NC-C^. A commonly used approach to rescue the infectivity of fluorescently-labeled viruses is to generate mixed virus particles through the co-transfection of virus producer cells with equal masses of plasmids encoding for virus containing wt and virus containing fluorescently-labeled proteins [4], [15]. One major limitation of this approach is that it results in a heterogeneous population of virus particles containing: (a) only wt proteins, (b) only fluorescently-labeled proteins and (c) a mixture of wt and fluorescently-labeled proteins. We have therefore adapted our previously described conditional co-transfection system [21] to produce fluorescently-labeled HIV-1 containing a known ratio of wt/TC tagged proteins. The production of viral particles is limited to cells that simultaneously express both wt and TC tagged proteins. This system takes advantage of the fact that both Gag and Rev are required for the formation of infectious viral particles.

**Figure 2:**
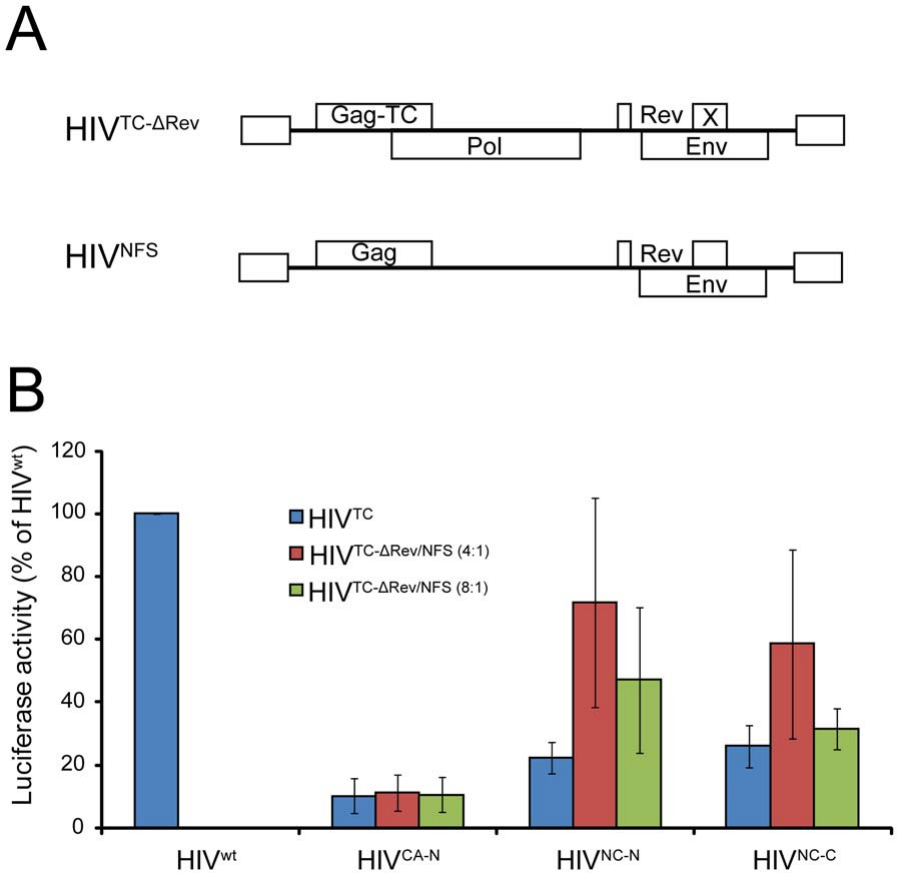
Improve the infectivity of the *gag*-TC viruses. (A) Schematic representation of proviral DNA constructs used in the study. The HIV^TC-ΔRev^ construct differs from HIV^TC^ by the introduction of an early termination codon and a frameshift into exon 2 of the Rev sequence, which was removed to inactivate Rev function. This mutation also affects Env expression. HIV^NFS^ is a full-length HIV-1 construct containing codon modifications in the -1 frameshift slippery sequences and the RNA pseudoknot in the *gag* reading frame to enable the expression of Gag but not Gag-Pol. (B) The capacity of HIV^TC-ΔRev/NFS^ to infect target cells was assessed by measuring luciferase activity in the indicator TZM-bl cells that have been infected with the indicated viruses. Error bars, s.d. are based on the averages of 3-5 independent experiments.

Firstly, the HIV^CA-N^, HIV^NC-N^, HIV^NC-C^ were modified to introduce an early termination codon and a frameshift into exon 2 of the Rev sequence (HIV^TC-ΔRev^) that inactivates Rev function and therefore prevents the nuclear export of the unspliced and single spliced HIV-1 mRNA. Thus, cells expressing only HIV^TC-ΔRev^ cannot generate virus particles. Secondly, we generated a full-length HIV-1 construct containing modifications at the -1 frameshift slippery sequence and the immediate downstream RNA pseudoknot that regulates Gag-Pol expression (non-frameshift HIV-1 or HIV^NFS^). These codon modifications altered the coding sequence in the *gag* reading frame without affecting the protein sequence enabling the expression of Gag but not Gag-Pol. As HIV^NFS^ lacks the capacity to produce HIV enzymatic proteins, cells expressing only HIV^NFS^ are unable to produce mature and infectious viral particles. The built-in constrains of HIV^TC-ΔRev^ and HIV^NFS^ ensure that fluorescent infectious HIV-1 can only be produced when both proviral DNA constructs are simultaneously expressed in the same cell. These HIV-1 constructs are depicted in Figure 2A.

As the natural ratio of Gag to Gag-Pol synthesis is 20∶1, the two plasmids were co-transfected at a ratio of 4 HIV^TC-ΔRev^ to 1 HIV^NFS^ or 8 HIV^TC-ΔRev^ to 1 HIV^NFS^ to generate a theoretical ratio of Gag to Gag-Pol of 25∶1 and 22.5∶1, respectively. These co-transfection ratios should also result in virus particles containing a ratio of TC tagged to wt Gag proteins of 80∶20 or 88.9∶11.1, respectively. Firstly, we compared the infectivity of these conditionally-produced fluorescent viruses with the original HIV^TC^ viruses (Figure 2B). HIV^NC-N-ΔRev/NFS(4∶1)^ and HIV^NC-C-ΔRev/NFS(4∶1)^ generated by co-transfection at a 4∶1 ratio showed an increase in infectivity (50% and 33%, respectively) when compared with the original HIV^NC-N^ and HIV^NC-C^ viruses. HIV^NC-N-ΔRev/NFS(8∶1)^ and HIV^NC-C-ΔRev/NFS(8∶1)^ generated by co-transfection at a 8∶1 ratio showed a less pronounced increase in infectivity (25% and 5%, respectively) when compared with the original HIV^NC-N^ and HIV^NC-C^ viruses. The reduced capacity of HIV^NC-ΔRev/NFS(8∶1)^ to rescue HIV infectivity when compared with HIV^NC-ΔRev/NFS(4∶1)^ is most likely due to an increase in the ratio of TC tagged to wt Gag incorporated into the viral particles. Secondly, we compared the infectivity of these conditionally-produced fluorescent viruses with HIV^wt^. HIV^NC-N-ΔRev/NFS(4∶1)^, HIV^NC-C-ΔRev/NFS(4∶1)^, HIV^NC-N-ΔRev/NFS(8∶1)^ and HIV^NC-C-ΔRev/NFS(8∶1)^ showed 72%, 59%, 47% and 31% infectivity when compared with HIV^wt^, respectively. In contrast, HIV^CA-N-ΔRev/NFS^ generated by co-transfection of proviral DNA at a 4∶1 or 8∶1 ratios did not show an increase in infectivity when compared with the original HIV^CA-N^. Our data shows that this conditional co-transfection system can be used to rescue the infectivity of selected HIV^TC^ viruses.

### Specific labeling of tetracysteine-tagged viral proteins

**Figure 3:**
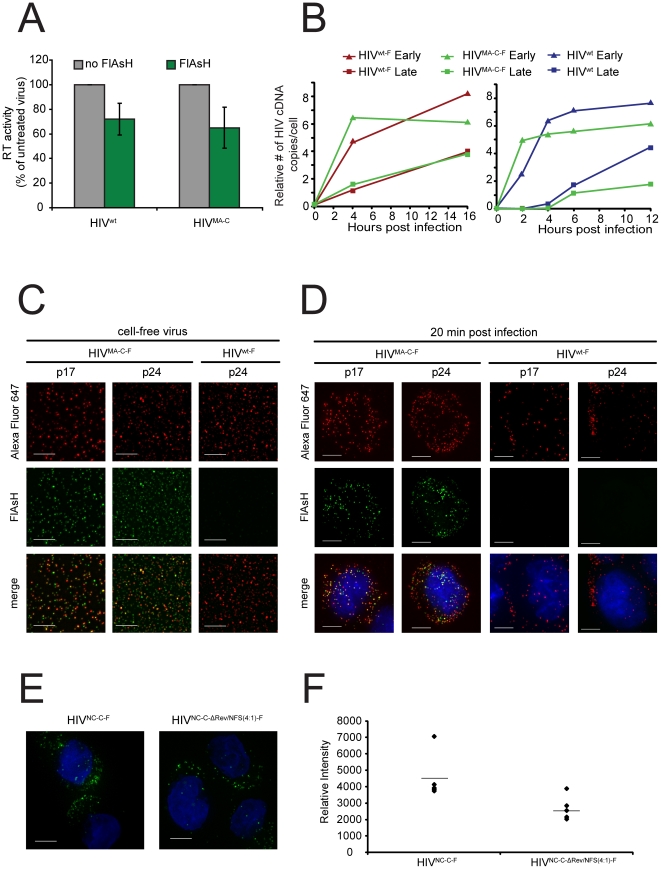
Labeling of tetracysteine-tagged matrix proteins of an infectious HIV-1 does not impact on virus function. (A) Production of HIV^wt^ and HIV^MA-C^ from 293Ts that have been untreated (no FlAsH) or incubated with 1 µM FlAsH-EDT_2_ (FlAsH). The level of virus production was measured with an *in vitro* reverse transcriptase assay that is specific for HIV-1 enzymatic activity. Data are representative of 2 independent experiments. Error bars represent technical replicates. (B) Quantitative PCR analysis of the kinetics of HIV-1 cDNA synthesis during an infection in MT-2 cells. Cells were infected with HIV^wt^ or with HIV^wt^ and HIV^MA-C^ produced in the presence of 1 µM FlAsH-EDT_2_ as described above (HIV^wt-F^ and HIV^MA-C-F^). Cells were harvested at the indicated time points post-infection and analyzed by quantitative PCR for both early ([-]ssDNA) and late (post second strand transfer) HIV-1 reverse transcription products. HIV-1 cDNA copies were normalized by the number of copies of the CCR5 gene in the host cells. Data are representative of 2 independent experiments. (C-D) Specificity of the labeling of tetracysteine-tagged virus with biarsenical dyes. (C) HIV^MA-C-F^ and HIV^wt-F^ (cell-free virus) were immunostained with HIV-1 p17-MA and p24-CA mAb followed by donkey anti-mouse Alexa Fluor 647. (D) MT-2 cells were fixed after 20 min of synchronized infection with HIV^MA-C-F^ or HIV^wt-F^ and immunostained with HIV-1 p17-MA and p24-CA monoclonal antibodies followed by donkey anti-mouse Alexa Fluor 647 secondary antibody. Nuclei were labeled with Hoechst. FlAsH is shown in green, Alexa Fluor 647 in red and nuclei in blue. The provided images were derived from a volume compression of a z stack of 10-38 images taken at a 0.2-µm step size. Scale bar, 5 µm. Images are representative of more than 3 independent experiments. (E) MT-2 cells were infected with HIV^NC-C^ or HIV^NC-C-ΔRev/NFS(4∶1)^ produced in the presence of 1 µM FlAsH-EDT_2_ as described above (HIV^NC-C-F^ or HIV^NC-C-ΔRev/NFS(4∶1)-F^, respectively). The infected cells were fixed after 4 h of synchronized infection, and the nuclei were labeled with Hoechst. FlAsH is shown in green and nuclei are shown in blue. The provided images were derived from a volume compression of a z stack of 26 images taken at a 0.5-µm step size. Scale bar, 5 µm. Images are representative of 2 independent experiments. (G) Quantification of the relative intensity of FlAsH staining using softWoRx software (Applied Precision, Issaquah, WA) following infection with HIV^NC-C-F^ or HIV^NC-C-ΔRev/NFS(4∶1)-F^. Each symbol represents a separate field from the same experiment. The black lines show the mean. Data are representative of 2 independent experiments.

The high level of background associated with the labeling of low-abundance TC tagged proteins has significantly limited its applicability in many virus systems. VSV-G pseudotyped viruses are often used to increase the signal to noise ratio. In order to establish a protocol where fluorescently labeled viruses can infect their natural target cells using native envelope glycoproteins, we have optimized a metabolic labeling system where TC-labeled viruses are produced in the presence of the biarsenical dye FlAsH. The excess dye and non-specifically labeled cellular debris were removed by sucrose density gradient centrifugation. A similar approach has been used recently for the labeling of a recombinant TC tagged-VSV [14], showing the general applicability and generic nature of this labeling approach. HIV^MA-C^ was chosen based on its wt level of infectivity. HIV^MA-C^ produced in the presence of the FlAsH dye is referred to as HIV^MA-C-F^. HIV^wt^ was also generated in parallel in the presence of the FlAsH dye (HIV^wt-F^) to evaluate the specificity of the labeling and the potential packaging of non-specifically labeled cellular proteins. HIV^wt-F^ and HIV^MA-C-F^ have indistinguishable levels of viral production (Figure 3A) and infectivity of a T-lymphocytic cell line (Figure 3B). Furthermore, HIV^MA-C-F^ also show comparable levels and kinetics of viral cDNA synthesis to HIV^wt^ (Figure 3B), illustrating that HIV^MA-C-F^ closely mimics the early events of HIV-1 replication. Our system results in a highly specific FlAsH labeling of TC tagged proteins in HIV^MA-C-F^ but not in HIV^wt^. The specificity of the FlAsH labeling was further confirmed by colocalization of HIV^MA-C-F^ signal and immunofluorescent staining with anti-MA or anti-CA monoclonal antibodies (Figure 3C–D). The same results were observed when using the biarsenical dye ReAsH (data not shown). Labeling of HIV^NC-C^ and HIV^NC-C-ΔRev/NFS(4∶1)^ with this metabolic labeling system shows that NC can also be visualized using the biarsenical-tetracysteine system (Figure 3E). Furthermore, HIV^NC-C-ΔRev/NFS(4∶1)-F^ showed only a minor decrease in fluorescence intensity when compared with the HIV^NC-C-F^, which is probably due to the fact that HIV^NC-C-ΔRev/NFS(4∶1)-F^ contains only 80% of TC-labeled NC proteins per virus particle in comparison with HIV^NC-C-F^ (Figure 3F).

## Discussion

The imaging of fluorescent-labeled viruses is limited to the visualization of a small number of viral proteins that can be fluorescently labeled without compromising virus infectivity. Due to its small size and versatility, the biarsenical-tetracysteine system is an attractive generic approach to fluorescently label viral proteins. The TC tag has been successfully used to label the HIV-1 MA [11], [12], HIV-1 IN [13] and VSV MA [5], [14] proteins. This study describes the labeling of HIV-1 structural, enzymatic and envelope proteins with a TC tag in the context of a full-length HIV-1 and its effect on virus infectivity.

The insertion of the TC tag in the V1 and V2 flexible loops of the Env gp120 showed surprisingly different results. While the insertion in the V2 loop completely abolished virus infectivity, the insertion in the V1 loop was less detrimental to virus infectivity. This suggests that flexibility is not the only requirement for the insertion of the TC tag and that the V1 loop but not the V2 loop of gp120 is a good candidate for the insertion of a TC tag. Furthermore, due to the limited number of Env molecules in the virion [22], the new generation of brighter biarsenical dyes such as Alexa Fluor 568-FlAsH may be more suitable for the visualization of TC tagged Env [23].

Insertion of the TC tag at the C-terminus of the PR did not affect the proteolytic processing of HIV-1 proteins, indicating that it can potentially be used for the visualization of the production, assembly, budding and maturation of HIV-1. On the other hand, insertion of the TC tag at the N- and C-terminus as well as in the flexible “thumb” subdomain of the RT dramatically affected virus production, entry and infectivity. This is not surprising due to the fact that the RT undergoes highly precise and complex structural rearrangements during reverse transcription that might be affected by the smallest modifications [24], [25]. A possible solution to this problem may be long-term culture of the HIV^RT^ in lymphocytes to introduce compensatory mutations and therefore restore virus infectivity. Introduction of the TC tag at the N-terminus of the IN abolished HIV-1 infectivity, which indicates that as described previously [13], the C-terminus of IN is more permissive to the introduction of the TC tag.

We confirm that the TC tag can be inserted at the C-terminus of the MA protein without affecting virus infectivity [11], [12]. Similarly to what has been described previously for the cyclophilin-binding loop of CA [15], insertion of the TC tag at both the N- and C-terminus of the CA protein completely abolished virus infectivity, suggesting that the stability of the highly ordered CA lattice might be compromised by the TC tag. The most encouraging result from this analysis is that the insertion of the TC tag at either the N- or C-terminus of the NC protein only partially affected virus infectivity. Using a conditional co-transfection system we were able to rescue the infectivity of both the HIV^NC-N^ and HIV^NC-C^ to 72% and 59% of the HIV^wt^ infectivity while retaining enough TC tagged NC proteins for visualization by fluorescence microscopy. These are the first infectious HIV-1 containing fluorescently-labeled NC proteins that will allow the visualization of the intracellular trafficking of the HIV-1 genome-NC complexes in live cells. Furthermore, the approaches described in this study can be applied to other virus systems. The genetic variances found within any group of viruses suggest that it is highly likely that a segment within the virus genome will be able to accommodate the six amino acids TC tag without compromising virus infectivity.

Using HIV^MA^ as a model system, we have improved the biarsenical-TC labeling system for the visualization of viral proteins during the early events of a natural virus infection. This generic protocol is built on three premises: firstly, the viral genome must contain segments that are sufficiently flexible for the insertion of a six amino acids TC tag without compromising the biological function of the protein and the infectivity of the virus; secondly, multiple copies of the TC tagged protein must be incorporated into the virion during assembly; thirdly, any non-specifically labeled cellular debris and non-incorporated free biarsenical dye must be readily removed from the labeled viruses through virus purification procedures.

Furthermore, it is imperative to generate a wild-type virus in parallel with the TC tagged virus in the presence of the biarsenical dye in order to evaluate the specificity of the labeling and the potential packaging of non-specifically labeled cellular proteins. By using a metabolic labeling approach that can easily be adapted to other viruses, we have bypassed the requirement for high concentrations of reducing agents during labeling. Using appropriate and specific virus purification procedures (such as sucrose density gradient centrifugation), we have found that non-specifically labeled cellular debris and excess dye can be efficiently removed, which leads to a substantial decrease in background fluorescence.

In conclusion, this study identifies novel tools for the imaging of HIV-1 proteins during its natural infection process. Furthermore, this study describes generic approaches to label viral proteins with a TC tag without compromising virus infectivity as well as a specific and generic method to improve the labeling of TC tagged viral proteins with biarsenical dyes in the context of an infectious virus.

## Methods

### Cells

293T cells were maintained in Dulbecco's modified Eagle medium/high modified (with 4500 mg/l dextrose and 4 mM L-glutamine) medium (DMEM; Invitrogen, Mount Waverley, Victoria, Australia), supplemented with 10% (vol/vol) heat-inactivated cosmic calf serum (CCS; Hyclone, Tauranga, New Zealand), 100 U/ml of penicillin and 100 µg/ml of streptomycin (Invitrogen). MT-2 cells (obtained through the AIDS Research and Reference Reagent Program, Division of AIDS, NIAID, NIH from D. Richman) [26], [27] were cultured in Rosewell Park Memorial Institute (RPMI) 1640 medium (Invitrogen) supplemented with 10% vol/vol heat-inactivated fetal calf serum (FCS; Invitrogen) and penicillin/streptomycin. TZM-bl cells (obtained through the AIDS Research and Reference Reagent Program, Division of AIDS, NIAID, NIH from Dr. John C. Kappes, Dr. Xiaoyun Wu and Tranzyme Inc.) are derived from the HeLa cell line and stably express high levels of CD4, CXCR4, and CCR5 on the cell surface and have integrated copies of the luciferase and β-galactosidase genes under control of the HIV-1 promoter. TZM-bl cells were cultured in DMEM supplemented with 10% CCS and penicillin/streptomycin. Peripheral Blood Mononuclear Cells (PBMCs) were isolated from buffy packs (supplied by the Red Cross Blood Bank, Melbourne) using Ficoll-Hypaque density gradient centrifugation (Amersham Pharmacia Biotech, Uppsala, Sweden). PBMCs were then stimulated with 10 µg/ml phytohemagglutinin (PHA; Remel, Lenexa, KS, USA) for 3 days and maintained in RPMI 1640 medium supplemented with 10% CCS, 2 mM L-glutamine (Invitrogen), 50 µg/ml gentamycin (Pfizer, Bentley, WA, Australia) and 10 U/ml of human interleukin-2 (IL-2; Roche, Mannheim, Germany).

### Viruses

The pNL4-3 proviral DNA (NIH AIDS Research & Reference Reagent Program, Dr. Malcolm Martin [28]) contains the NL4-3 infectious molecular clone of HIV-1. The HIV-1 constructs with a tetracysteine (TC) tag were generated using stitch PCR mutagenesis on the full-length pDRNL. Sequence-specific primers were used to introduce the TC motif at the sites indicated in Figure 1A and Table 1. The region of interest was then amplified using HIV-1 specific primers and subcloned back into the pDRNL backbone using the appropriate restriction sites. The primers used in this study are described in Table 2. In the HIV^MA-C^, HIV^CA-N^, HIV^CA-C^, HIV^NC-N^, HIV^NC-C^, HIV^PR-C^, HIV^RT-N^, HIV^RT-C^, HIV^RN-N^, HIV^RN-C^ and HIV^IN-N^ constructs, the TC tag was inserted five amino acids downstream and/or upstream of each of the protease cleavage sites in the Gag or Gag-Pol precursor protein and a *Nae I* restriction site was introduced downstream or upstream of the TC tag to improve screening. In addition, the first or last five amino acids of the viral protein coding sequence were repeated downstream or upstream of the TC tag to maintain the viral protease cleavage sites.

**Table 2:** Primers used for site-directed mutagenesis.

Name	Primers containing the tetracysteine motif (5′ ->3′)	
HIV^MA-C^	FW	gccggctgctgccgcgagtgctgcgtcagccaaaattaccctatagtgcagaacctccaggg
	RW	gtaattttggctgacgcagcactcgcggcagcagccggcgtaattttggctgacctggctg
HIV^CA-N^	FW	cctatagtgcagaactgctgccgcgagtgctgcgccggccctatagtgcagaacctccaggg
	RW	gccggcgcagcactcgcggcagcagttctgcactatagggtaattttggctgacctggc
HIV^CA-C^	FW	gccggctgctgccgcgagtgctgcaaagcaagagttttggctgaagcaatgagccaagtaac
	RW	caaaactcttgctttgcagcactcgcggcagcagccggccaaaactcttgctttatggccggg
HIV^NC-N^	FW	atacagaaaggcaattgctgccgcgagtgctgcgccggcatacagaaaggcaattttaggaacc
	RW	gccggcgcagcactcgcggcagcaattgcctttctgtatcattatggtagctggatttgttacttg
HIV^NC-C^	FW	gccggctgctgccgcgagtgctgcgagagacaggctaattttttagggaagatctggccttcc
	RW	attagcctgtctctcgcagcactcgcggcagcagccggcattagcctgtctctcagtacaatctttc
HIV^PR-C^	FW	gccggctgctgcggcccatgctgctgcactttaaattttcccattagtcctattgagactg
	RW	aaaatttaaagtgcagcagcatgggccgcagcagccggcaaaatttaaagtgcagccaatctg
HIV^RT-N^	FW	cccattagtcctatttgctgcggcccatgctgcgccggccccattagtcctattgagactg
	RW	gccggcgcagcatgggccgcagcaaataggactaatgggaaaatttaaagtgcagccaatctg
HIV^RT-C^	FW	gccggctgctgcggcccatgctgcggagcagaaactttctatgtagatggggcagccaatagg
	RW	gaaagtttctgctccgcagcatgggccgcagcagccggcgaaagtttctgctcctattatggg
HIV^RTt1^	FW	tgctgccccgggtgctgcgacagctggactgtcaatgac
	RW	gcagcacccggggcagcacttttctggcagcactatagg
HIV^RTt2^	FW	tgctgccccgggtgctgcccactaacagaagaagcagagc
	RW	gcagcacccggggcagcatactacttctgttagtgctttgg
HIV^RN-N^	FW	tatgtagatggggcatgctgcggcccatgctgcgccggctatgtagatggggcagccaatagg
	RW	gccggcgcagcatgggccgcagcatgccccatctacatagaaagtttctgctcctattatggg
HIV^RN-C^	FW	gccggctgctgcggcccatgctgcatcaggaaagtactatttttagatggaatagataaggcc
	RW	tagtactttcctgatgcagcatgggccgcagcagccggctagtactttcctgattccagc
HIV^IN-N^	FW	tttttagatggaatatgctgcggcccatgctgcgccggctttttagatggaatagataaggcc
	RW	gccggcgcagcatgggccgcagcatattccatctaaaaatagtactttcctgattccagc
HIV^Env-V1^	FW	tacctgctgtccaggatgttgcaatagtagtagcgggagaatg
	RW	tattgcaacatcctggacagcaggtattagtatcattcttcaaatc
HIV^Env-V2^	FW	taattgctgtccaggatgttgcaccagctataggttgataagttg
	RW	tggtgcaacatcctggacagcaattatctattggtactatatcaag

Name	HIV-1 NL4.3 specific primers (5′ ->3′)	
HIV 631	FW	tggcgcccgaacagggac
NL 3700	RW	ggataggtggattatgtgtcatccatcc
NL 3538	FW	cacagtggggggacatcaagcagcca
NL 4266	RW	tggccttcccttgtgggaagg
NL 1878	FW	ctgaagcaatgagccaagtaac
NL 2500	RW	ttgacaggtgtaggtcctac
NL 4373	FW	gacaacaactccctctcagaagcaggagccgata
NL 5176	RW	catgctacactggaatattgctgg
HIV Age I	FW	taaggcaattatgtaaacttcttagg
HIV Not I	RW	catattgtgagtctgtcactatg
HIV Not I	FW	gaagttatggtaccagttagaga
HIV Pflm I	RW	ggtatttcttatagcagattctg

The HIV^TC-ΔRev^ plasmid differs from HIV^TC^ by the introduction of an early termination codon and a frameshift into exon 2 of the Rev sequence, which inactivates Rev function. The Rev inactivation mutation was achieved by incorporation of a early stop codon in the *BamHI* site. The HIV^NFS^ plasmid was constructed by replacing the wt sequence with 5′ CTTCCTCGGGAAG ATATGGCCATCACACAAAGGTAGACCT 3′ between DNA nucleotides 4240-4266 employing the ApaI and BclI sites of the HIV-1 molecular clone [29].

HIV-1 particles were produced by poly(ethylenimine) (PEI; Polysciences Inc., Warrington, PA, USA) transfection of 293T cells cultured in 10-cm plates with: 3.5 µg pDRNL or HIV^TC^ plasmids to generate unlabeled viruses; 2.5 µg pDRNL or HIV^TC^ plasmids and 1 µg pMM310 β-lactamase-Vpr plasmid (kindly donated by M. Miller, Merck Research Laboratories) to generate β-lactamase-Vpr-labeled viruses; 5 µg HIV^TC-ΔRev^ plasmids and 2.5, 1.25 or 0.63 µg HIV^NFS^ plasmid to generate HIV^TC-ΔRev/NFS^ viruses. Forty hours post-transfection supernatant from 293T cells was harvested, purified, filtered and virus production quantified by micro-reverse transcriptase (RT) assay. Briefly, a sample of the culture supernatant was mixed with an equal volume of 0.3% vol/vol Nonidet P-40, followed by addition of RT reaction cocktail containing the template primer poly(rA)-(dT)_15_ (GE Healthcare, Rydalmere, Australia) and [α^33^P]-dTTP (PerkinElmer, Waltham, MA, USA). Following incubation for 3 h at 37°C, RT activity was determined by the level of [α^33^P]-dTTP using a TopCount NXT^TM^ Microplate Scintillation and Luminescence Counter (Perkin Elmer, Glen Waverley, Australia). Viral particles were concentrated by ultracentrifugation through a 20% sucrose cushion at 100,000 *g* for 1 h at 4°C using an L-90 ultracentrifuge (SW 41 rotor; Beckman, Fullerton, CA, USA) and virus pellets were resuspended in 1x phosphate buffered saline (PBS; Invitrogen) and quantified using a HIV-1 antigen (p24 CA) Micro enzyme-linked immunosorbent assay (ELISA) (Vironostika: Biomerieux, Boxtel, The Netherlands).

### Metabolic labeling of virus with biarsenical dyes

Eight hours after transfection the virus producer cells were incubated with 1 µM FlAsH-EDT_2_ (Invitrogen) premixed with 12.5 µM 1,2-Ethanedithiol (EDT; Sigma-Aldrich, Sydney, Australia) and virus production was allowed to proceed for 40 h. Viral particles were isolated, purified, filtrated and concentrated as described above. Non-incorporated free biarsenical dye was removed from the virions by sucrose density gradient centrifugation. Briefly, biarsenical dye-labeled viral particles were layered on top of sucrose density gradients prepared in PBS in 2.5% increments ranging from 30 to 55% sucrose and centrifuged for 16 h at 100,000 × *g* (SW41 rotor; L-90 Ultracentrifuge). Afterwards, gradient fractions were collected, a micro-RT assay was used to determine the location of the virion particles in the gradient fractions, the virus-containing fractions were pooled together and the virus was re-concentrated by ultracentrifugation and quantified by (p24-CA) Micro ELISA.

### Protein isolation and western blot analysis

Intracellular viral protein was isolated from transfected 293T cells by washing cells twice with PBS then lysing with Tris-buffered saline (TBS) lysis buffer containing 1% Nonidet P-40, 20 mM phenylmethylsulfonylfluoride (PMSF), 1 µM pepstatin and 1 µM leupeptin. Cell lysates were freeze-thawed in liquid nitrogen and then clarified by centrifugation. Concentrated virions were obtained by ultracentrifugation as described above and lysed with TBS lysis buffer. Western blot analysis was carried out using pooled sera from HIV-1 infected patients to detect total HIV-1 proteins.

### β-lactamase assay

One million MT-2 cells were infected with HIV^wt/BlaM-Vpr^ and HIV^MA/BlaM-Vpr^ viruses (normalized to 100 ng of p24) for 1 h at 37°C, 5% CO_2_, washed and loaded with the β-lactamase substrate CCF2-AM (GeneBLAzer in vivo Detection Kit, Invitrogen) for 7 h at RT. Afterwards, the cells were washed and fixed with 4% formaldehyde (Polysciences, Warrington, PA) in 0.1 M Pipes buffer, pH 6.8. The extent of virus-cell fusion was determined from the ratio of blue (440–480 nm) and green (518–538 nm) emission of CCF2-AM upon exciting the cells at 405–415 nm using a Fluorescence-Activated Cell Sorter (FACScan^TM^ Flow Cytometer; BD, North Ryde, Australia) and analyzed using FlowJo software (FlowJo, Ashland, OR).

### Luciferase assay

TZM-bl cells were placed in 96-well plates at a concentration of 2E+04 cells/well and incubated for 24 h at 37°C, 5% CO2. Afterwards the culture medium was replaced by culture medium containing 10 µg/µl diethylaminoethyl-dextran (DEAE-dextran) and the cells infected with 2-fold serial dilutions of pDRNL or HIV^TC^ viruses. After 48 h incubation at 37°C, 5% CO2 the medium was aspirated and 35 µl of cell culture lysis reagent (Promega, Wisconsin, USA) was added to each well. 5 µl of each sample was mixed with 25 µl of Luciferase Assay Substrate (Promega) and luminescence was measured using the FLUOstar OPTIMA multi-detector reader (BMG Labtech).

### Replication kinetics in PBMCs

Sample virus stocks with equivalent levels of RT activity, as determined by a micro-RT assay, were added to 1×10^5^ PBMCs in 96-well tissue culture plates. Eight 10-fold serial dilutions of each virus were tested in triplicate. Supernatants were collected on days 3, 7, 10 and 14 post-infection and virus infectivity was measured by monitoring the production of viral RT activity by using a micro-RT assay.

### Infection of lymphoid cells

Synchronized infections were performed as described previously [30]. MT-2 cells were spinoculated with virus at 17°C for 2 h at 1,200 × *g*. Afterwards, the cells were washed twice with PBS to remove unbound virus and incubated with warm media at 37°C, 5% CO_2_ for 20 min to initiate infection. Afterwards, the cells were washed, treated with 2 mg/ml of protease from *Streptomyces griseus* (pronase E; Sigma) for 10 min on ice and washed extensively with PBS containing 20% FCS. The cells were then fixed with 4% formaldehyde in 0.1 M Pipes buffer, pH 6.8 or incubated at 37°C, 5% CO_2_ for 4 h before fixation and cytospined into glass slides. Cell-free viruses (same batch as used for the infection of lymphoid cells) were fixed with 4% formaldehyde (Polysciences) in 0.1 M Pipes buffer, pH 6.8, spread on glass slides, incubated at 4°C for 16 h and washed twice with PBS.

### Immunofluorescence staining

Cells and virus were permeabilized and stained with mouse anti-matrix (SVM-33) antibody (MH-SVM33C9, ATCC, Manassas, VA (Akzo Nobel N.V.) and/or mouse anti-capsid (AG3.0) antibody (NIH AIDS Research & Reference Reagent Program, Dr. Jonathan Allan) [31] and goat Cy5-conjugated anti-mouse secondary antibody (Jackson ImmunoResearch, USA). After staining, the cells were counterstained with Hoechst 33258 (Invitrogen). Samples were mounted in Fluoromount-G (Electron Microscopy Sciences, Hatfield, PA) and images were captured in a z series on a charge-coupled device (CCD) camera (CoolSnap HQ; Photometrics, Tucson, AZ) through a 100x 1.4 numerical aperture (NA) oil immersion lens on a DeltaVision microscope (Applied Precision, Issaquah, WA) and deconvolved using softWoRx deconvolution software (Applied Precision).

### Quantitative PCR for HIV-1 reverse transcription products

Quantification of HIV-1 reverse transcription products and standardization of cell numbers was performed using quantitative PCR. Concentrated virus stocks were treated with 90 U/ml of Benzonase (Sigma) for 15 min at 37°C before infection to remove any contaminating plasmid DNA from the transfection procedure. A heat-inactivated virus control (2 h at 56°C) was used to confirm efficient removal of plasmid DNA for each sample. MT-2 cells were infected with equivalent amounts of virus as determined by a HIV-1 antigen (p24-CA) Micro ELISA by spinoculation at 17°C for 2 h at 1,200 × *g* [30]. Cells were then washed with warm PBS and then culture in fresh medium at 37°C. Cells were harvested at various time points post-infection and lysed in PCR lysis buffer containing 10 mM Tris [pH 8.0], 50 mM KCl with 0.5% vol/vol Triton-X100, 0.5% vol/vol NP-40 and 75 mg/ml proteinase K (Roche). Samples were incubated at 56°C for 2 h before the proteinase K was inactivated at 95°C for 10 min, samples were then stored at –20°C. Quantitative PCR was performed on an MX3000P QPCR machine (Stratagene). Each PCR reaction contained 1x Brilliant II SYBR Green Master mix (Stratagene), 400 nM each primer and 5 µl of cell lysates (1∶10 dilution) in a 15 µl reaction volume. The HIV-1 specific primers M667 (5′-GGCTAACTAGGGAACCCACTG-3′) and AA55 (5′-CTGCTAGAGATTTTCCACACTGAC-3′) were used to detect early HIV-1 cDNA ([-]strong-stop DNA). The HIV-1 specific primers M667 (5′-GGCTAACTAGGGAACCCACTG-3′) and M661 (5′-CCTGCGTCGAGAGATCTCCTCTGG-3′) were used to detect late HIV-1 reverse transcription products (post 2nd strand transfer). HIV-1 PCR conditions were an initial denaturation at 95°C for 15 min followed by 40 rounds of cycling at 95°C for 10 s, then 60°C for 30 s. Cell numbers were standardized for the human CCR5 gene using the primers LK46 (sense; 5′-GCTGTGTTTGCGTCTCTCCCAGGA-3′) and LK47 (antisense; 5′-CTCACAGCCCTGTGCCTCTTCTTC-3′). CCR5 PCR conditions were an initial denaturation at 95°C for 10 min followed by 40 rounds of cycling at 95°C for 20 s, 58°C for 40 s and 72°C for 40 s.
